# The Association Between an Individual’s Local Food Environment and Diet Quality among Postpartum Women Living in Rural Bangladesh

**DOI:** 10.1016/j.cdnut.2025.106011

**Published:** 2025-03-24

**Authors:** Alexandra L Bellows, Andrew Thorne-Lyman, Saijuddin Shaikh, Md Tanvir Islam, Shahnaj Parvin, Rezwanul Haque, Monica M Pasqualino, Frank Curriero, Hasmot Ali, Alain B Labrique, Md Iqbal Hossain, Amanda C Palmer

**Affiliations:** 1 Department of International Health, Johns Hopkins Bloomberg School of Public Health, Baltimore, MD, United States; 2 JiVitA Maternal and Child Health and Nutrition Research Project, Gaibandha, Bangladesh; 3 Department of Epidemiology, Johns Hopkins Bloomberg School of Public Health, Baltimore, MD, United States; 4 icddr,b, Dhaka, Bangladesh

**Keywords:** food environment, dietary diversity, diet quality, Bangladesh, markets

## Abstract

**Background:**

The food environment is a driver of the double burden of malnutrition, influencing dietary intake by increasing or restricting access to foods.

**Objectives:**

The objective of this study was to assess the association between geospatial food environment indicators and the diet quality among postpartum women in rural Bangladesh.

**Methods:**

Participants were women of infants enrolled in a cluster-randomized controlled trial from 2018 to 2020. Food vendor availability was defined as the number of food vendors within a specific household radius, and proximity was defined as the distance to the nearest vendor. Dietary intake was measured using a 7-d food frequency questionnaire collected at 3 mo, 6 mo, and 12 mo postpartum. Our primary outcome was nonstarchy staple food variety scores (FVS). Secondary outcomes included dietary diversity scores and individual food group consumption. To assess the association between food environment indicators and diet quality indicators, we fit linear regression models for the FVS outcome, Poisson regression models for the dietary diversity outcome, and logistic regression models for individual food group outcomes.

**Results:**

A total of 5064 women were included in this analysis. Women reported consuming an average of 9.7 (standard deviation: 3.7) nonstarchy staple foods in the previous week. Women who lived in households with the highest market availability (≥7 markets within 1600 m) had an average of 0.84-unit (95% confidence interval: 0.53, 1.16) higher FVS compared with those in households with the lowest market availability (≤ 2 markets) (*P* < 0.001). Geospatial food environment indicators were not significantly associated with the odds of consuming less healthy food options.

**Conclusions:**

We found a positive relationship between market availability and diet quality for postpartum women in rural Bangladesh. However, more research is needed to understand which components of the food environment are associated with increased consumption of less healthy foods.

## Introduction

In many South Asian countries, diets are shifting from more traditional diets to diets high in saturated fat, added sugar, and ultraprocessed foods, a phenomenon known as the nutrition transition [1]. Low diet quality is associated with premature mortality and is one of the leading causes of mortality worldwide [2,3]. Poor-quality diets that are insufficient in energy and essential nutrients can lead to micronutrient deficiencies and undernutrition. A high prevalence of undernutrition is observed among women, particularly those of reproductive age and in the postpartum period, because of increased biological demand for nutrients due to menstruation, pregnancy, or lactation and sometimes unequal allocation of food within a household [4]. What constitutes a healthy diet may vary based on context, culture, and individual characteristics, but generally, healthy diets include a diverse selection of nutrient-rich foods and a low-to-moderate intake of foods high in saturated fat, added sugars, and sodium [5].

The food environment, the places where people obtain their food [6,7], has been identified as a common driver of the double burden of malnutrition, particularly food environments where nutrient-dense food is unaffordable or scarcely available, and less healthy food options are increasingly more affordable and available to consumers [8,9]. In many countries in South Asia and sub-Saharan Africa, food environments are transitioning from more traditional food vendors to more modern retailers (e.g., supermarkets) and increasing access and affordability of both healthy and less healthy food options [6,10].

Most studies assessing the food environment have focused on high-income country (HIC) contexts, but the greatest opportunity is in countries with transitioning food systems, where there are increasing concerns about the double burden of malnutrition at the community, household, and/or individual level [11,12]. Yet, the relationship between food environments and diet quality in this context is not well understood, particularly in rural areas where undernutrition remains a prominent concern and prevalences of overweight and obesity are rising [13]. Results from HICs may not be transferable to other settings due to large differences in the types of food retailers available and health outcomes of interest [6,7].

In Bangladesh, the prevalence of underweight among women of reproductive age has decreased significantly from 43% to 12% from 2000 to 2022 [14]. Moreover, in that same period, the prevalences of overweight [BMI (in kg/m^2^] 25 to <30] and obesity (BMI ≥30) among women of reproductive age have nearly quadrupled, from 6% to 26.3% and 2% to 8%, respectively [14]. In this study, we aimed to characterize the food environment in a rural area of Bangladesh and assess which aspects of the food environment are associated with higher consumption of nutrient-rich foods and limited consumption of less healthy food options for postpartum women. We hypothesized that *1*) women with greater market availability and proximity would have a more diverse diet and *2*) women with greater grocery shop availability and proximity would have higher odds of consuming less healthy food options.

## Methods

### Study population and setting

Participants included in this analysis were women enrolled in the Protein Plus trial, a cluster-randomized controlled infant feeding trial testing the independent and combined effects of protein supplementation and enteric disease control, from September 2018 to March 2020 (clinicaltrials.gov: NCT03683667). This trial was conducted at the JiVitA Research Site, located in Gaibandha District in northwestern Bangladesh. The JiVitA Research Site was established in 2000 by researchers at Johns Hopkins University and has hosted multiple cluster-randomized community trials and observational studies. The research site is ∼430 km^2^, with a population of about 630,000 individuals [15].

Researchers identified women and infants for the Protein Plus trial via ongoing pregnancy and birth surveillance activities carried out for the mCARE-II trial, testing the efficacy of a digital health intervention on maternal and infant outcomes (clinicaltrials.gov: NCT02909179). Women were eligible for mCARE-II if they were married, lived with their husbands, consented to participation in pregnancy surveillance, and became pregnant during the study recruitment period. At 3 mo postpartum, women and infant dyads enrolled in mCARE-II were invited to participate in the Protein Plus trial. Trained field staff obtained written consent from all participants. Women were followed from 3 mo postpartum to 18 mo postpartum. Study protocols for the mCARE-II trial were approved by the Johns Hopkins Bloomberg School of Public Health Institutional Review Board, Baltimore, Maryland, and the Bangladesh Medical Research Council, Dhaka, Bangladesh. Study protocols for the Protein Plus trial were approved by the Johns Hopkins Bloomberg School of Public Health Institutional Review Board, Baltimore, Maryland, and the Research and Ethics Review Committees of the International Center for Diarrhoeal Disease Research, Dhaka, Bangladesh.

### Household and maternal assessments

Trained interviewers for the mCARE-II trial collected information on maternal age, maternal education, head of household education, household members’ occupations, number of people residing in the household, household assets, and housing characteristics at the time of enrollment.

For the Protein Plus trial, data on enrolled women were collected at 3 mo, 6 mo, 12 mo, and 18 mo. Maternal surveys included information on maternal health and food behaviors and practices. At 3-mo, 6-mo, and 12-mo postpartum, participating mothers were asked to complete a 7-d food frequency questionnaire (FFQ). Formative research informed the selection of commonly consumed food items that contributed to diet quality and micronutrient intake. The FFQ included 50 food items or food groups (Supplemental Table 1). Women were asked in Bangla, “How many times in the past week have you consumed [food item]?” Rice was not included in the questionnaire due to previously observed minimal variability in rice consumption within the study population. Most households in this area consume rice at least twice a day [16,17]. To reduce participant burden, the FFQ asked about the consumption of dark leafy greens, ruminant meat (goat, lamb, or beef), and poultry (chicken, duck, or goose) as food groups. Because of the rolling enrollment of the trial, dietary data were collected during all seasons. At the 12-mo visit, the questionnaire included a subset of questions from the women’s empowerment in agriculture index [18]. For a sensitivity analysis described in further detail below, we focused on a question in the decision-making module that asked women, “Can you, on your own, always, sometimes, or never decide to buy small amounts of food like rice, vegetables, dal, fish, etc.?” We used this question to approximate the food decision-making power of women in our analysis.

### Geospatial data collection

Field staff collected household geospatial coordinates during demographic household data collection. In addition, trained field staff conducted a landmark geospatial food environment survey in December 2020. This survey collected global positioning system (GPS) coordinates of stationary food vendors (markets, grocery shops, tea shops), schools, and clinics using GPS-enabled tablet devices. Markets were defined as a group of 5 or more shops open daily. In our definition of market, we included both formal (containing permanent concrete shops) and informal markets (no concrete shops). Grocery shops were defined as small shops that are open daily and sell groceries [e.g., raw rice (chaat), wheat flours, salt, dal, jaggery, oil, soap, cigarettes, and packaged foods] and were not part of a group of 5 or more similar shops. Grocery shops in this area do not typically sell fresh produce, animal-source foods, or prepared foods. The field site was divided into sectors (an administrative unit used for cluster randomization), and interviewers were instructed to collect GPS coordinates from all eligible establishments within their assigned sector. All spatially referenced data were entered into ArcPro [19] for data management.

### Statistical analysis

Analysis was conducted using ArcGIS Pro 2.5 [19] and The R Statistical Computing Environment (version 4.2.0) [20]. Women were included in the analysis if they had completed ≥2 dietary assessments not during Ramadan and resided in a household with GPS coordinates available. We excluded women whose age at enrollment was missing. In addition, we selected 1 woman per household to be included in the analysis. In the case where multiple women from the same household were enrolled, we randomly selected 1 woman from each household.

To measure socioeconomic status, we used household assets and housing characteristics to create a living standard index (LSI) using principal component analysis [21,22]. For this analysis, we categorized LSI into quintiles. Household food insecurity scores were calculated by summing responses from 9 food behavior questions. Participants were asked a series of questions about how often they experienced challenges with food access in the prior 6 mo using a 5-point Likert scale (1 = Never, 5 = Most days of the week). We categorized households that scored 9 (indicating they responded “Never” to all questions) as food secure. Households that scored 10–15 and >15 were categorized as having mild-to-moderate food insecurity and severe food insecurity, respectively [23]. The season of data collection was defined using the 6 seasons of the Bangladeshi calendar: winter (mid-December to mid-February), spring (mid-February to mid-April), summer (mid-April to mid-June), early monsoon (mid-June to mid-August), late monsoon (mid-August to mid-October), and autumn (mid-October to mid-December) [17].

To assess usual intake, we averaged the consumption of food items or food groups at 3, 6, and 12 mo. We excluded dietary assessments conducted during the month of Ramadan because diet during this time may not reflect usual consumption [24]. Our primary outcome of interest was nonstarchy staple food variety scores (FVS). We defined this as the average number of nonstarchy staple foods items or groups consumed in the last week. This score does not include less healthy food options such as vegetable oil, soda, sweet yogurt, sugar cane, cake, biscuits, mishti (a Bengali sweet), chocolate, candy, ice cream, salty snacks, and foods fried in oil. We created a dichotomous consumption variable for each food item. A woman received a value of 0 for each food item if they reported consuming the food item < 1 time per week and a value of 1 if they reported consuming the food item 1 or more times per week. We calculated FVS by summing the dichotomous variables for all food items. We chose FVS as the primary outcome of interest over dietary diversity scores (DDS) because the larger range of the FVS allowed us to treat the outcome as a continuous variable, making the results more easily interpretable.

Our secondary outcomes of interest include DDS and consumption of individual food groups. DDS was calculated by summing the number of food groups consumed using the minimum dietary diversity score for women (MDD-W) food group categorization: cereals, flesh foods, pulses & beans, dark leafy green vegetables, other vitamin A-rich vegetables, dairy, eggs, other fruits, other vegetables, and nuts and seeds (Supplemental Table 1) [25]. DDS scores are normally calculated by summing the number of food groups consumed in the last 24 h [25]. Because our study collected dietary intake over the last 7 d, we considered women to have consumed a food group if they reported consuming foods within that food group 3 or more times in the previous week. DDS ranged from 0 to 10.

To define the consumption of individual food groups, we again used food group categorization from MDD-W guidelines [25]. However, we separated flesh foods into 3 food groups due to the high fish consumption in our population: seafood (fresh fish, dried fish, and prawns), ruminant meat, and poultry. In addition, we assessed the consumption of less healthy food options, a category not traditionally included in the MDD-W food group guidelines. Less healthy food options included soda, sweet yogurt, sugar cane, cake, biscuits, mishti (a Bengali sweet), chocolate, candy, ice cream, salty snacks, and foods fried in oil. We categorized the consumption of individual food groups as a dichotomous variable. Women who consumed foods within a food group an average of 3 or more times in the previous week were given the value of 1, and women who consumed foods within that food group an average of fewer than 3 times in the previous week were given a value of zero.

Geospatial data were imported into ArcPro and projected to EPSG: 3106 (Gulshan 303 Bangladesh Transverse Mercator) to calculate the distance in meters from households. Food vendors included in this analysis were markets and grocery shops. We calculated food vendor proximity and availability. Proximity was defined as the distance to the nearest market or grocery shop from households, which was measured by Euclidean distance. Availability was defined as the density of markets within 1600 m or the density of grocery shops within 400 m and 1600 m of households. We chose these boundaries to reflect immediate and village-level proximity to households [26]. Food environment variables were calculated at the household level.

We used multiple regression analyses to examine the association between food availability and proximity indicators (i.e., the density of markets or grocery shops and distance to the nearest market or grocery shop, respectively) with dietary intake indicators (i.e., FVS, DDS, and consumption of specific food groups). Linear regression models were fit for the continuous FVS outcomes, Poisson regression for the discrete count DDS outcomes (counts ranging from 0 to 10), and logistic regression for the binary consumption of individual food group outcomes. Coefficients from the Poisson regression analysis are reported as incidence rate ratios (IRRs), and coefficients from the logistic regression analysis are reported as odds ratios (ORs). Proximity exposure variables were scaled to 100 m units for interpretability purposes. Availability markets and grocery shops were categorized using quartiles based on the distribution of data. Regression analyses were adjusted for potential confounding factors selected based on a priori knowledge and covariates used in previous studies examining the relationship between food environment and dietary intake [26,27]. Model 1 adjusted for maternal age. Model 2 adjusted for maternal age, LSI (quintiles), maternal education (categorical), number of people residing in the household, and season of the 3-mo maternal survey. Model 3 adjusted for covariates included in model 2 and distance to or density of other food vendors. For example, models with distance to the nearest market as the main exposure of interest were adjusted for the distance to the nearest grocery shop. Covariates included in the model were tested for collinearity using variance inflation factors (VIFs). Variables with a VIF >10 were excluded from the model. Maternal occupation was excluded from models because of high collinearity with maternal education. Model residuals were checked for fit.

To test the robustness of our results, we ran sensitivity analyses excluding observations that were identified to have a high influence on each model. We calculated Cook’s distance for each observation and defined high influence as an observation having a Cook’s distance >4/*n* [28]. For models with FVS and DDS as the outcome, a *P* value of <0.05 was considered significant. For models assessing the consumption of individual food groups as an outcome, we used a Bonferroni-adjusted significance threshold of *P* < 0.005 (0.05/10) to account for multiple comparisons.

We conducted a final set of sensitivity analyses to see if our results were confounded by differences in women’s ability to make decisions on purchasing small food items such as rice, vegetables, dal, or fish. In these models, we excluded women who reported never being able to make decisions regarding the purchase of small food items.

## Results

The Protein Plus trial enrolled 5891 women and their infants. For this analysis, we excluded 376 women because they did not have >1 dietary assessment that was not collected during Ramadan. Another 383 women were excluded because geospatial coordinates for their primary residence were not available. Finally, 28 women were excluded because of missing maternal age at enrollment. After selecting 1 woman per household, an additional 40 women were removed from our analysis (Supplemental Figure 1). We compared the demographic characteristics of women included in and excluded from this analysis. Women who were excluded from the analysis were slightly younger, but otherwise, the 2 groups were comparable (Supplemental Table 2). A total of 5064 women were included in our analysis, with 3274 (64.7%) and 1790 (35.3%) women having 3 and 2 dietary assessments, respectively. Characteristics of women and households are presented in Table 1. The average age at enrollment was 23.3 (SD: 5.5) y. The majority of women (69%) had some primary education, with 19% of women reporting having passed the secondary school certificate or reporting some form of secondary education. The average household size was 4.5 (SD: 1.9) people. Most (61%) women reported no formal occupation, whereas two-thirds of households reported the head of household’s occupation to be a laborer or a business owner. Only 14% of households reported head of household’s primary occupation as farming. Food insecurity was reported by 32% of households, with the majority of these households reporting mild or moderate food insecurity.

**TABLE 1:** Baseline characteristics of women and households enrolled in the Protein Plus trial (*n* = 5064).

	*n*	Mean ± SD or *n* (%)
Maternal characteristics		
Age	5064	23.3 ± 5.5
Maternal education	5059	
No schooling		628 (12.4)
Class 1–9		3490 (68.9)
SSC passed		304 (6.0)
11 y and above		637 (12.6)
Maternal occupation	5063	
No occupation		3093 (61.1)
Farmer/sharecropper		77 (1.5)
Laborer		253 (5.0)
Business owner		1400 (27.7)
Private service		199 (3.9)
Other		41 (0.8)
Household characteristics		
Household size	5060	4.5 ± 1.9
Household head’s occupation	4952	
No occupation		61 (1.2)
Farmer/sharecropper		700 (13.8)
Laborer		1642 (32.4)
Business owner		1670 (33.0)
Private service		756 (14.9)
Other		123 (2.4)
Food security^1^	4655	
Food secure (HFI ≤ 9)		3087 (66.3)
Mild or moderate food insecurity (HFI 10–15)		1440 (30.9)
Severe food insecurity (HFI ≥ 16)		128 (2.7)

Table includes only women who completed either 2 or 3 dietary assessments over the course of the trial and live in households with available geospatial coordinates.

Abbreviations: SSC, secondary school certificate; HFI, household food insecurity.

1 Household food insecurity scores were calculated by summing responses from 9 food behavior questions. Participants were asked about frequency of behavior in the last 6 mo using a 5-point Likert scale (1 = Never, 5 = Most days of the week).

**FIGURE 1:**
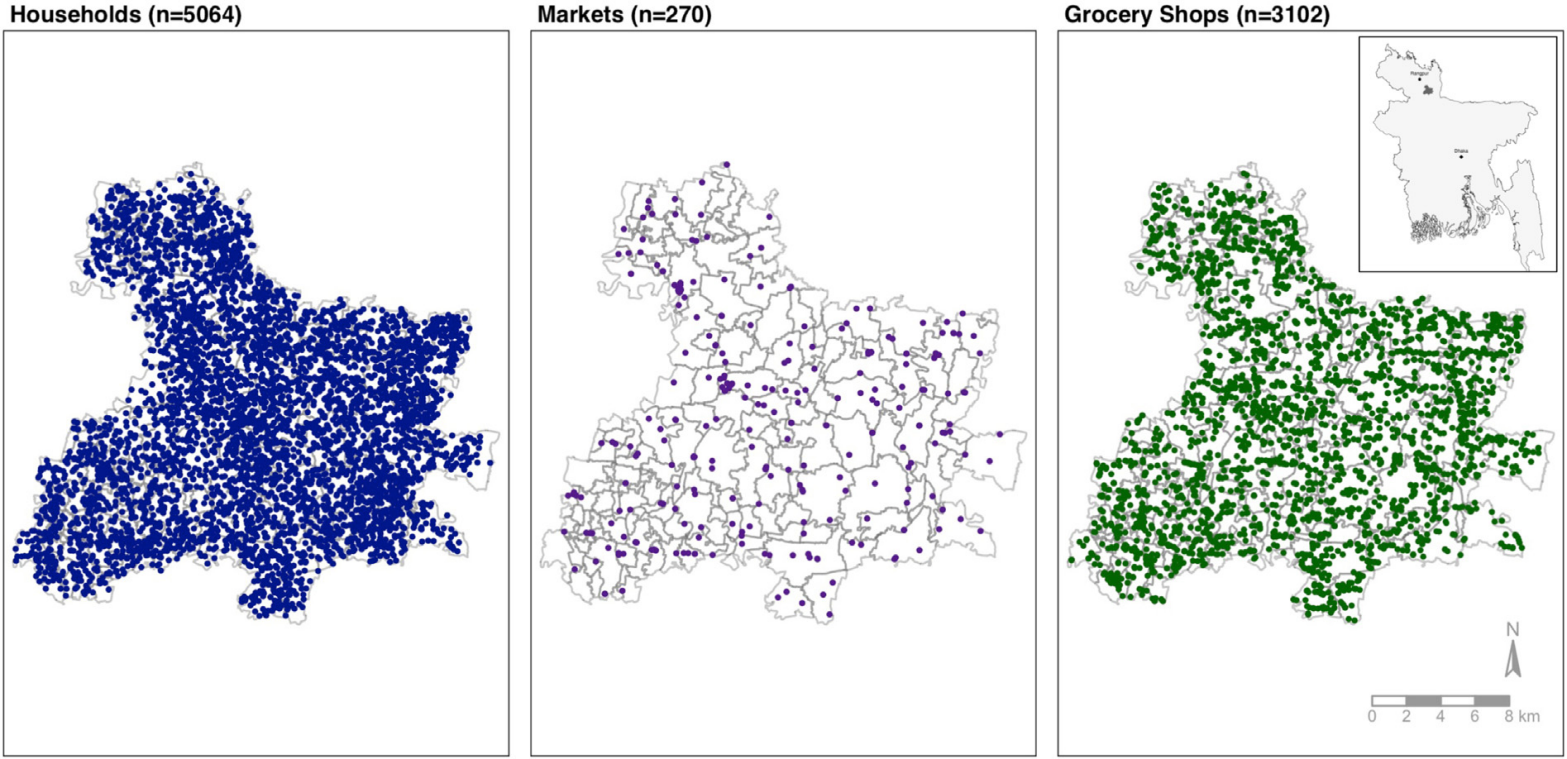
Map of households, markets, and grocery shops in the JiVitA study area during the Protein Plus trial. Household geospatial data were collected at baseline enrollment in the study area from 2018 to 2020. Markets and grocery shop geospatial data were collected during the geospatial survey in December 2020. Household map is displayed soley to show density of the area; locations of households have been spatially jittered by a random factor to retain anonymity.

In the study area, 270 markets and 3102 grocery shops were identified during the geospatial survey (Figure 1). The average Euclidean distance to the nearest market for households was 626.7 m (median: 592.0), and the average number of markets within a 1600 m radius of a household was 5.1 (median: 5.0). The majority of households did not have a market within a 400 m radius (71.0%). The average distance to the nearest grocery shop for households was 176.9 m (median: 149.7). The average number of grocery shops within a 400 m and 1600 m radius of households was 4.6 and 56.0 (medians: 4.0 and 55.0), respectively (Table 2).

**TABLE 2:** Descriptive statistics for distance to nearest food vendor and density of food vendors for households enrolled in Protein Plus trial (*n* = 5064).

	Mean ± SD	Median (Q1, Q3)
Markets		
Distance to nearest market^1^ (m)	626.72 ± 344.00	592.00 (359.43, 851.56)
Density of markets within 400 m^2^	0.44 ± 0.91	0.00 (0.00, 1.00)
Density of markets within 1600 m	5.06 ± 3.05	5.00 (3.00, 6.00)
Grocery shops		
Distance to nearest grocery shop (m)	176.88 ± 124.22	149.65 (82.80, 242.63)
Density of grocery shops within 400 m	4.64 ± 3.20	4.00 (2.00, 6.00)
Density of grocery shop within 1600 m	55.96 ± 16.51	55.00 (45.00, 68.00)

1 Distance to nearest food vendor was measured in straight line Euclidean distance from households.

2 Density measured as the number of food vendors within 400 m and 1600 m of households.

Women reported consuming an average of 9.7 (SD: 3.7) nonstarchy staple foods in the previous week (FVS). The most commonly consumed nonstarchy staple foods were eggplant (5.0 times/wk), large fish (3.4 times/wk), small fish (2.0 times/wk), biscuits/cake (2.0 times/wk), and eggs (1.9 times/wk). The mean DDS was 4.2 out of 10 food groups (median: 4.0, IQR: 3–5). Cereals, other vegetables, and seafood were the most commonly consumed food groups among participants (Table 3). In addition, 47.5% of women reported consuming less healthy food options an average of 3 or more times in the previous week.

**TABLE 3:** Descriptive statistics for frequency of dietary outcomes^1^ from a 7 d recall period among postpartum women (*n* = 5064).

	Mean ± SD	Median (IQR)	Range
Diet quality scores			
Food variety score^2^	9.7 ± 3.7	9.0 (7.0–12.0)	1.0–25.0
Dietary diversity score^3^	4.2 ± 1.4	4.0 (3.0–5.0)	1.0–10.0
Food groups			
Cereals	17.8 ± 4.2	19.0 (15.3–21.0)	0.0–33.0
Other vegetables^4^	10.2 ± 6.1	9.3 (5.7–13.5)	0.0–56.0
Seafood	6.2 ± 4.0	5.3 (3.3–8.3)	0.0–28.0
Unhealthy foods^5^	3.5 ± 3.2	2.7 (1.0–5.0)	0.0–21.7
Pulses	3.1 ± 2.5	2.5 (1.3–4.0)	0.0–20.5
Other fruits^6^	1.9 ± 2.3	1.0 (0.3–2.7)	0.0–22.5
Eggs	1.9 ± 1.7	1.3 (0.7–2.7)	0.0–14.0
Dairy	1.6 ± 2.2	0.5 (0.0–2.7)	0.0–25.5
DGLV	1.5 ± 1.4	1.3 (0.7–2.0)	0.0–14.0
Ruminant meat	1.0 ± 1.5	0.5 (0.0–1.3)	0.0–16.0
Other vitamin A-rich fruits and vegetables^7^	1.0 ± 1.5	0.3 (0.0–1.3)	0.0–17.5
Poultry	1.0 ± 1.2	0.7 (0.0–1.5)	0.0–10.0
Nuts and seeds	0.1 ± 0.4	0.0 (0.0–0.0)	0.0–4.7

Abbreviation: DGLV, dark leafy green vegetables; MDD-W, minimum dietary diversity score for women.

1 Averaged from 3 time periods: 3 mo, 6 mo, and 12 mo postpartum.

2 Food variety scores are defined as the average number of nonstarchy staple foods items or groups consumed in the last week excluding sweet and salty snacks and sugary sweetened beverages.

3 Dietary diversity scores were defined as the number of food groups consumed ≥3 times on average in the 7-d recall period. Scores range from 1 to 10. Food groups defined using MDD-W guidelines: cereals, flesh foods, pulses & beans, dark leafy green vegetables, other vitamin A-rich vegetables, dairy, eggs, other vegetables, other fruits, and nuts and seeds.

4 Other vegetables consist of bitter gourd, okra, taro stems, pointed gourd, gourd, radish, ripe tomato, eggplant, cucumber, jamur/jam, pickles, cabbage, and cauliflower.

5 Unhealthy foods consist of soda, sweet yogurt, sugar cane, cake/biscuits, mishti, chocolate/candy, ice cream, salty snacks, and food fried in oil.

6 Other fruits consist of ripe banana, ripe jackfruit, guava, jalpai/aamra, watermelon, pineapple, green fruit, pomello/ jambura/orange.

7 Other vitamin A-rich fruits and vegetables consist of ripe mango, ripe papaya, and ripe pumpkin.

### Diet quality scores

We found a positive association between the availability of markets and FVS. Our results indicate a dose–response pattern with higher coefficients associated with greater market availability and are consistent across all market quartiles. Women who lived in households with the highest market density (≥7 markets within a 1600 m radius) had an average of 0.84-unit [95% confidence interval (CI): 0.53, 1.16] higher FVS compared with women who lived in households with the lowest market density (≤ 2 markets within 1600 m) (*P* < 0.001) (Table 4). After controlling for sociodemographic confounders, market proximity and proximity or availability of grocery shops were not associated with FVS. Sensitivity analysis indicated these results were consistent even when excluding women who reported having no decision over food purchases (Supplemental Table 3). Consistent with findings from the FVS models, women who lived in households with the highest market density (≥ 7 markets within 1600 m) had significantly higher DDS compared with women who lived in households with the lowest market density (≤ 2 markets within 1600 m) (IRR: 1.07; 95% CI: 1.03, 1.12; *P* < 0.01). After controlling for sociodemographic confounders, market proximity and proximity or availability of grocery shops were not associated with DDS (Supplemental Tables 4 and 5).

**TABLE 4:** Association between food environment indicators and food variety scores (using a 7-d recall period) among postpartum women (*n* = 5064).

	Model 1^1^		Model 2^2^		Model 3^3^	
	Estimate (95% CI)	*P* value	Estimate (95% CI)	*P* value	Estimate (95% CI)	*P* value
Markets						
Distance to nearest market^4^ (100 m)	−0.04(−0.07, −0.01)	0.01	−0.02(−0.05, 0.01)	0.11	−0.02(−0.05, 0.01)	0.12
Density of markets within 1600 m^5^						
0–2 markets	Ref		Ref		Ref	
3–4 markets	0.39 (0.09, 0.68)	0.01	0.38 (0.10, 0.65)	<0.01	0.42 (0.14, 0.70)	<0.01
5–6 markets	0.49 (0.19, 0.79)	<0.01	0.46 (0.18, 0.74)	<0.01	0.52 (0.24, 0.80)	<0.001
7 ≥ markets	0.81 (0.50, 1.12)	<0.001	0.72 (0.44, 1.01)	<0.001	0.84 (0.52, 1.15)	<0.001
Grocery shops						
Distance to nearest grocery shop (100 m)	0.05 (−0.03, 0.13)	0.24	0.05 (−0.02, 0.13)	0.19	0.05 (−0.03, 0.12)	0.20
Density of grocery shops within 400 m						
0–1 grocery shops	Ref		Ref		Ref	
2–3 grocery shops	−0.39 (−0.72, −0.06)	0.02	−0.38 (−0.68, −0.08)	0.01	−0.38 (−0.68, −0.08)	0.01
4–5 grocery shops	0.07 (−0.26, 0.39)	0.69	0.01 (−0.29, 0.31)	0.96	0.01 (−0.29, 0.30)	0.97
6 ≥ grocery shops	−0.21 (−0.52, 0.10)	0.19	−0.26 (−0.54, 0.03)	0.08	−0.28 (–0.57, 0.01)	0.06
Density of grocery shops within 1600 m						
0–44 grocery shops	Ref		Ref		Ref	
45–54 grocery shops	−0.03 (−0.32, 0.26)	0.83	−0.06 (−0.33, 0.21)	0.65	−0.18 (−0.46, 0.09)	0.18
55–67 grocery shops	0.06 (−0.22, 0.35)	0.66	−0.04 (−0.30, 0.23)	0.79	−0.22 (−0.50, 0.05)	0.11
68 ≥ grocery shops	0.03 (−0.26, 0.31)	0.86	0.06 (−0.21, 0.32)	0.67	−0.26 (−0.55, 0.04)	0.09

Food variety scores are defined as the average number of nonstarchy staple foods items or groups consumed in the last week excluding sweet & salty snacks and sugary sweetened beverages. Scores range from 1 to 25.

Abbreviation: CI, confidence interval.

1 Model 1 controls for maternal age.

2 Model 2 controls for maternal age, living standards index (categorical), maternal education (categorical), number of people in household, and season of 3-mo dietary recall (categorical).

3 Model 3 controls for model 2 covariates + distance/density of grocery shops (market models) or markets (grocery shop models).

4 Distance variables were scaled to 100 m units. Negative numbers indicate households living further from food vendors have on average lower food variety scores.

5 Density variables were categorized using quartiles based on the distribution of data.

### Individual food group consumption

As shown in Table 5, multivariable regression model estimates suggested that women who lived in households with the highest density of markets had a 46% increased odds of consuming pulses (OR: 1.46; 95% CI: 1.20, 1.77; *P* < 0.001) and 44% increased odds of consuming foods in the “other fruit” category (OR: 1.44; 95% CI: 1.15, 1.81; *P* = 0.001) compared with women who lived in households with the lowest market density. After adjusting the significance level to account for testing multiple outcomes, market proximity or availability was not significantly associated with the odds of consuming “other vegetables,” dark leafy green vegetables, “other vitamin A-rich fruits and vegetables,” seafood, eggs, dairy, ruminant meat, or poultry (Table 5).

**TABLE 5:** The association between food environment indicators and consumption of individual food groups (≥ 3 times over 7 d) among postpartum women (*n* = 5064).

Food group	Other vegetables^1^		Pulses		Other fruit^2^		DGLV		Other VitA F&V^3^	
	Odds ratio (95% CI)	*P* value	Odds ratio (95% CI)	*P* value	Odds ratio (95% CI)	*P* value	Odds ratio (95% CI)	*P* value	Odds ratio (95% CI)	*P* value
Distance to nearest market^4^ (100 m)	1.03 (1.00, 1.06)	0.09	0.99 (0.97, 1.01)	0.19	0.99 (0.97, 1.01)	0.29	0.98 (0.95, 1.00)	0.06	0.97 (0.94, 0.99)	0.02
Density of markets within 1600 m^5^										
0–2 markets	Ref		Ref		Ref		Ref		Ref	
3–4 markets	1.16 (0.86, 1.56)	0.33	1.25 (1.06, 1.49)	<0.01	1.14 (0.93, 1.40)	0.2	0.94 (0.73, 1.21)	0.64	1.07 (0.79, 1.46)	0.63
5–6 markets	1.25 (0.92, 1.70)	0.16	1.33 (1.12, 1.58)	<0.01	1.04 (0.85, 1.29)	0.69	1.23 (0.96, 1.58)	0.1	1.32 (0.97, 1.79)	0.08
7 ≥ markets	1.22 (0.86, 1.72)	0.27	1.45 (1.20, 1.76)	<0.001	1.44 (1.15, 1.81)	0.001	1.34 (1.02, 1.76)	0.04	1.41 (1.01, 1.98)	0.05

Food group	Seafood		Eggs		Dairy		Ruminant meat		Poultry	
	Odds ratio (95% CI)	*P* value	Odds ratio (95% CI)	*P* value	Odds ratio (95% CI)	*P* value	Odds ratio (95% CI)	*P* value	Odds ratio (95% CI)	*P* value
Distance to nearest market (100 m)	1.01 (0.99, 1.03)	0.32	1.01 (0.99, 1.03)	0.56	0.99 (0.97, 1.01)	0.24	0.99 (0.97, 1.02)	0.64	0.98 (0.95, 1.01)	0.22
Density of markets within 1600 m										
0–2 markets	Ref		Ref		Ref		Ref		Ref	
3–4 markets	1.22 (0.99, 1.50)	0.06	1.12 (0.92, 1.37)	0.27	1.16 (0.94, 1.42)	0.16	1.00 (0.75, 1.32)	0.98	1.42 (1.03, 1.99)	0.04
5–6 markets	1.21 (0.98, 1.49)	0.08	1.07 (0.87, 1.32)	0.50	1.16 (0.94, 1.43)	0.17	0.69 (0.51, 0.93)	0.01	1.23 (0.88, 1.75)	0.22
7 ≥ markets	1.17 (0.93, 1.48)	0.18	1.23 (0.99, 1.54)	0.07	1.23 (0.98, 1.55)	0.07	0.94 (0.69, 1.29)	0.72	1.10 (0.76, 1.60)	0.61

Outcome variable for each model was binary. Participants were considered to have consumed specified food group if they reported consuming foods within that food ≥3 times on average in the 7-d recall period. All models control for maternal age, living standard index (categorical), maternal education (categorical), number of people in household, and season of 3-mo dietary assessment (categorical). We used Bonferroni-adjusted significance threshold of *P <* 0.005 (0.05/10) to account for multiple comparisons.

Abbreviations: DGLV, dark green leafy vegetables, Other VitA F&V, other vitamin A fruits and vegetables.

1 Other vegetables consist of bitter gourd, okra, taro stems, pointed gourd, gourd, radish, ripe tomato, eggplant, cucumber, jamur/jam, pickles, cabbage, and cauliflower.

2 Other fruit consist of ripe banana, ripe jackfruit, guava, jalpai/aamra, watermelon, pineapple, green fruit, and pomello/ jambura/orange.

3 Other vitamin A-rich fruits and vegetables consist of ripe mango, ripe papaya, and ripe pumpkin.

4 Distance variables were scaled to 100 m units. Negative numbers indicate households living further from food vendors have on average lower food variety scores.

5 Density variables were categorized using quartiles based on the distribution of data.

The distance to the nearest market had a slight inverse association with the odds of consuming less healthy food options. For every 100 m increase in distance to the nearest market, there was a 2% decrease in the odds of consuming less healthy food options (OR: 0.98; 95% CI: 0.97, 1.00; *P* = 0.05). On the other hand, the density of markets, distance to the nearest grocery shop, and density of grocery shops were not significantly associated with the odds of consuming fewer healthy food options among women in this study (Table 6). We found similar results in a sensitivity analysis which excluded women who reported having no decision over food purchases (Supplemental Table 6).

**TABLE 6:** The association between food environment indicators and consumption of less healthy food options (≥ 3 times over 7 d) among postpartum women (*n* = 5064).

	Model 1^1^		Model 2^2^		Model 3^3^	
	Odds ratio (95% CI)	*P* value	Odds ratio (95% CI)	*P* value	Odds ratio (95% CI)	*P* value
Markets						
Distance to nearest market^4^ (100 m)	0.98 (0.96, 1.00)	0.02	0.98 (0.97, 1.00)	0.05	0.98 (0.97, 1.00)	0.05
Density of markets within 1600 m^5^						
0–2 markets	Ref		Ref		Ref	
3–4 markets	1.06 (0.90, 1.25)	0.48	1.08 (0.91, 1.28)	0.40	1.09 (0.92, 1.30)	0.33
5–6 markets	1.09 (0.93, 1.29)	0.29	1.10 (0.92, 1.30)	0.30	1.11 (0.93, 1.33)	0.23
7 ≥ markets	1.05 (0.89, 1.25)	0.57	1.01 (0.85, 1.21)	0.91	1.03 (0.85, 1.26)	0.74
Grocery shops						
Distance to nearest grocery shop (100 m)	1.01 (0.97, 1.06)	0.60	1.01 (0.97, 1.06)	0.54	1.01 (0.97, 1.06)	0.56
Density of grocery shops within 400 m						
0–1 grocery shops	Ref		Ref		Ref	
2–3 grocery shops	0.98 (0.82, 1.18)	0.89	0.99 (0.82, 1.19)	0.92	0.99 (0.82, 1.19)	0.92
4–5 grocery shops	0.93 (0.78, 1.11)	0.45	0.90 (0.75, 1.09)	0.29	0.90 (0.75, 1.09)	0.28
6 ≥ grocery shops	0.96 (0.81, 1.14)	0.62	0.94 (0.79, 1.13)	0.51	0.93 (0.78, 1.11)	0.43
Density of grocery shops within 1600 m						
0–44 grocery shops	Ref		Ref		Ref	
45–54 grocery shops	1.04 (0.89, 1.22)	0.59	1.04 (0.88, 1.23)	0.64	1.03 (0.87, 1.22)	0.75
55–67 grocery shops	0.96 (0.82, 1.12)	0.61	0.92 (0.78, 1.08)	0.30	0.90 (0.76, 1.07)	0.24
68 ≥ grocery shops	0.97 (0.83, 1.14)	0.73	0.97 (0.82, 1.14)	0.73	0.97 (0.81, 1.17)	0.76

Less healthy food consumption is defined as consumption of less healthy food options ≥3 times on average in the 7-d recall period. Less healthy foods options were defined as soda, sweet yogurt, sugar cane, cake/biscuits, mishti, chocolate/candy, ice cream, salty snacks, and food fried in oil.

1 Model 1 controls of maternal age.

2 Model 2 controls for maternal age, living standards index (categorical), maternal education (categorical), number of people in household, and season of 3-mo dietary assessment (categorical).

3 Model 3 controls for model 2 covariates + distance/ density of grocery shops (market models) or markets (grocery shop models).

4 Distance variables were scaled to 100 m units. Negative numbers indicate households living further from food vendors have on average lower food variety scores.

5 Density variables were categorized using quartiles based on distribution of data.

## Discussion

In this rural region of Bangladesh, we found a positive relationship between market availability and diet quality for postpartum women. The diets of postpartum women consisted of mostly starchy staples, seafood, and nonleafy green vegetables. Nearly half of the women in this study reported consuming less healthy food options, such as biscuits, cake, and other packaged foods, >3 times per week. In this cross-sectional analysis of food environment indicators and dietary intake, we found a marginally positive association between the density of markets close to the home and women’s dietary diversity, based on FVS and DDS. Women in households with higher market density were more likely to consume pulses and non-vitamin A-rich fruits. On the other hand, distance to the nearest market and grocery shop density were not associated with dietary intake outcomes.

The relationship between markets and diets has been primarily explored among smallholder farmer households in low and middle-income country (LMIC) settings. Most studies have assessed the relationship between market access—measured as distance to the nearest market—and individual dietary diversity, a proxy measure for inadequate micronutrient intakes and risk of deficiency, or household dietary diversity, a proxy measure for food access [29]. In contrast to our results which found no association, many of these studies find a significant inverse relationship between distance to the nearest market and diet indicators, although the magnitude of these associations tended to be small [[30], [31], [32], [33]]. In addition, even though our study area is considered rural in the context of Bangladesh, the majority of households in our study area were not smallholder farms. An analysis of pooled data from Indonesia, Kenya, Ethiopia, and Malawi found that a 10 km increase in distance to the nearest market among smallholder farmers was associated with a −0.01 decrease in household dietary diversity. However, when stratified by country, the effect was only statistically significant for Malawi [31]. Additionally, a study in rural Tanzania found that distance to the nearest market had a small inverse association with diet quality among women of reproductive age. A 1 km increase in distance to the nearest market was associated with a 0.27 decrease in diet quality score [30].

Our results may differ from other studies on market access in rural LMIC settings because of high population density and better access to markets. A review of geospatial measures of the food environment noted that proximity metrics, such as distance to the nearest food vendor, to measure food access are more useful in areas with lower concentrations of vendors and households [34]. Although the JiVitA Research Site is considered a rural area of Bangladesh, Bangladesh is one of the most densely populated countries in the world [35], and the average population density was estimated to be 1352.8 people/km^2^ in this setting [36]. In addition, the average distance to the nearest market was ∼650 m, which is much closer than in other studies in rural LMIC settings, where the average distance to the nearest market ranged from 1.1 km in Tanzania [30] to 63 km in Ethiopia [31].

Our results found a positive association between market availability and both diet quality scores, FVS and DDS. Our results are consistent with other studies in HIC settings, where most studies found null results when examining proximity measured as the distance to the nearest vendor [37], whereas other studies found that market density rather than the distance to the nearest market is associated with higher diet quality [38,39]. When access to markets is not an issue, increased density of markets may facilitate diverse food acquisition by increasing the number of foods available. Competition between different vendors may also lead to lower food prices, thereby increasing the amount and number of foods a household could purchase for consumption. A qualitative study conducted among residents in peri-urban India found that food price and affordability were key drivers of food acquisition, with residents noting that they would be willing to travel further for better prices [40]. These findings are consistent with those in HICs, where studies have found that consumers do not typically shop at the nearest supermarket [[41], [42], [43], [44]].

Certain foods, such as fruits, pulses, and animal-source foods, may be more likely purchased at a market compared with others. Therefore, we explored the relationship between consumption of different food groups and market indicators. Although the odds of consuming most food groups increased for women living in households with the highest market density, only consumption of pulses and non-vitamin A-rich fruits was statistically significant. The association between access to and consumption of fruit and vegetables is well documented, particularly in HICs, where diet-related noncommunicable diseases are predominant. A systematic review of studies conducted primarily in HICs found a positive association with increased access to and increased intake of fruits and vegetables [45]. This relationship may differ in an LMIC setting due to higher consumption of vegetables, the importance of price, and other nonmarket sources of fruits and vegetables. The relationship between market availability and “other vegetable” consumption may be null in our population because of the near-ubiquitous consumption of foods in the “other vegetable” group. For dark green leafy vegetables and other vitamin A-rich fruits and vegetables, individual characteristics, such as maternal education and socioeconomic status (SES), appear to be stronger predictors for consumption than market access, indicating that purchasing power may be more important. In addition, the relationship between markets and fruit and vegetable consumption may be weak due to other sources of fruits and vegetables in this study area. Home gardens and homestead agriculture can be sources of fruits and vegetables [46,47]. Although we did not capture information on home gardening practices in this study, data collected in the same time period for another study at our research site indicated that 94% of households had a home garden (West, unpublished data). Additionally, other studies have found that informal vendors such as mobile vendors are important suppliers of fruits and vegetables [27]. An unfortunate limitation of many geospatial food environment surveys is the difficulty of capturing these informal vendors [48].

Market density was not significantly associated with the consumption of any animal-source foods. This is particularly surprising for fish, poultry, and ruminant meat, as these foods are less likely to be produced at home for consumption. A previous analysis using data from the Protein Plus trial assessed the relationship between infant dietary intake and household animal production and found that household animal production of eggs and dairy was associated with infant consumption of these foods, whereas most households reported purchasing fish, poultry, and ruminant meat [49]. In our analysis, maternal education and SES status had the strongest relationship with animal-source food consumption. Women with the highest levels of education and SES had 2–3 times higher odds of consuming animal-source foods, indicating that the price and affordability of these products are significant determinants of consumption. These results are consistent with other studies in Bangladesh that have found strong associations between animal-source food consumption, wealth, and maternal education levels [50].

An interesting and unexpected finding of this study is the lack of association between the density of grocery shops and consumption of less healthy food options. Grocery shops in the study area sell predominately packaged foods, including biscuits—one of the most commonly consumed foods reported by women. As food systems transition, processed and packaged foods are becoming more prevalent even in rural areas [[51], [52], [53], [54]]. A study in Viet Nam found that packaged foods such as cakes and biscuits were less expensive in rural areas compared with urban areas and rural residents had a high average consumption of these foods compared with urban residents [55]. The authors hypothesized that the presence of these foods in traditional convenience stores close to rural residents’ homes may have increased the consumption of these foods. One potential explanation for the lack of association between grocery shop density and the consumption of less healthy food options in our study is the uniformly high density of these shops in the study area, likely nullifying any potential relationship with dietary intake. In addition, individual characteristics such as SES and education level are associated with less healthy food consumption, a finding that is also consistent with previous findings regarding adolescents’ diets at the JiVitA Research Site [16].

Our study is one of the few in a rural South Asian context to measure the association between built food environment and diet quality, focusing on both adequacy and moderation components of the diet. A further strength was the collection of dietary data from a large sample of women at 3 different time points over a 9-mo period, which allowed us to better characterize usual long-term intake of foods. We also benefited from a comprehensive geospatial survey of the study area. However, there are several limitations. First, our characterization of the built food environment was based solely on the geospatial coordinates of food vendors. Food vendor proximity and availability is only 1 component of the food environment, which includes other external factors such as price, food availability, and vendor infrastructure, and internal factors such as affordability and convenience [7]. Unmeasured components of the food environment such as price and food availability within stores are likely to be important determinants of food acquisition and consumption. Second, we measured diet using a qualitative 7-d FFQ. This questionnaire did not capture the quantity of food consumed or consumption of foods not listed in the survey. Third, although all dietary data collection was completed prior to the COVID-19 pandemic, our geospatial data collection was carried out at the end of the parent trial, up to 2 y after dietary data collection. Geospatial data collection also coincided with the COVID-19 pandemic. In this rural area of Bangladesh, the COVID-19 pandemic did not likely impact the geospatial measures of markets, although economic constraints and social distancing measures may have forced smaller shops, such as grocery shops, to close. However, given the saturation of grocery shops in this area, we believe that the lack of relationship between shops and food consumption is due to oversaturation rather than lack of access. There were very few participants who did not have a variety store within 400 m of their household (*n* = 305, 6%). In addition, COVID-19 pandemic most likely impacted the supply of food, particularly fresh produce, and animal-source foods at markets due to restrictions in movement across the country [56], but this would not impact the results from our study as we only assessed geospatial measures rather than food sold at each location.

Our study highlights several potential avenues to explore further the relationship between the food environment and diet quality. Although we found that increased market availability was associated with higher diet quality, our findings also emphasize the need for more in-depth research beyond the geospatial assessment of the food environment. We need more research using market surveys to assess the availability of different types of food and their affordability, qualitative interviews with women and key food systems stakeholders to better understand the perceived enablers and inhibitors within the food environment, and food systems mapping to understand the flow of food within this context.

## Author contributions

The authors’ responsibilities were as follows – MIH, ABL, ACP: designed the research; ALB, MMP, SS, MTI, HA, ABL: conducted the research; ALB, AT-L, FC: analyzed the data; ALB: wrote the manuscript; ALB, ACP: primary responsibility for final content; and all authors: read and approved the final manuscript.

## Data availability

Data described in the manuscript, code book, and analytic code will be made available on request pending application and approval.

## Funding

ALB was supported by the Johns Hopkins Procter and Gamble Fellowship and Johns Hopkins Department of International Health Tuition Support. Funding for the original trial was provided by the Bill and Melinda Gates Foundation (OPP1163259). Under the grant conditions of the Bill and Melinda Gates Foundation, a Creative Commons Attribution 4.0 Generic License has already been assigned to the Author Accepted Manuscript version that might arise from this submission. The funders contributed to the trial’s design, but played no role in data collection, analysis, or preparation of the manuscript.

## Conflict of interest

ACP reports financial support was provided by Bill and Melinda Gates Foundation. ALB reports financial support was provided by Procter and Gamble Fellowship. The other authors declare that they have no known competing financial interests or personal relationships that could have appeared to influence the work reported in this article.
