# Supplementary material for: The Association Between an Individual’s Local Food Environment and Diet Quality among Postpartum Women Living in Rural Bangladesh
Source: Curr Dev Nutr. 2025 Mar 24;9(5):106011. doi: 10.1016/j.cdnut.2025.106011 (PMC12049991; doi:10.1016/j.cdnut.2025.106011)
Supplement: multimedia component 1 [file mmc1.docx]

# Supplemental Figures and Tables:

**Supplemental Table 1.** Food Group categorizations for JiVitA-6 Trial

| **Food Groups** | **Food Items from Protein Plus FFQ** |
| --- | --- |
| Grains, roots, and tubers | Wheat flour, potato (except sweet potato) |
| Other vegetables | Bitter gourd, okra, taro stems, pointed gourd, gourd, radish, ripe tomato, eggplant, cucumber, jamur/jam, pickles, cabbage, cauliflower |
| Seafood | Small fish, large fish, dried fish, shrimp |
| Less healthy food options | Soda, sweet yogurt, sugar cane, cake/biscuits, mishti, chocolate/candy, ice cream, salty snacks, food fried in oil, and chattu |
| Pulses | Dal/legumes/pulses, beans/peas |
| Eggs | Eggs (any source) |
| Dairy | Milk, sour yogurt, sweet yogurt |
| Other fruits | Ripe banana, ripe jackfruit, guava, jalpai/aamra, watermelon, pineapple, green fruit, pomello/ jambura/orange |
| Dark leafy greens and vegetables | Dark green leafy vegetables (any kind) |
| Ruminant Meat | Goat/lamb/beef |
| Poultry | Chicken/duck/goose |
| Other vitamin A-rich fruits and vegetables | Ripe mango, ripe papaya, ripe pumpkin |
| Nuts and seeds | Groundnut |
| FFQ: Food frequency questionnaire |  |

Supplemental Figure 1. Summary of participant flow for analytic subsample

| **Supplemental Table 2.** Baseline characteristics of women and households included (n=5,064) and excluded (n=827) from analytic subsample | | | | |
| --- | --- | --- | --- | --- |
|  | **Included** (n=5,064) | | **Excluded** (n=827) | |
|  | **n** | **Mean ± SD or n (%)** | **n** | **Mean ± SD or n (%)** |
| *Maternal Characteristics* |  |  |  |  |
| Age | 5064 | 23.3 ± 5.5 | 718 | 22.03 ± 5.11 |
| Maternal Education | 5059 |  |  |  |
| No schooling |  | 628 (12.4) | 737 | 58 (7.0) |
| Class 1-9 |  | 3490 (68.9) |  | 529 (64.0) |
| SSC passed |  | 304 (6.0) |  | 41 (5.0) |
| 11 years and above |  | 637 (12.6) |  | 109 (13.2) |
| Maternal Occupation | 5063 |  | 737 |  |
| No occupation |  | 3093 (61.1) |  | 491 (59.4) |
| Farmer/sharecropper |  | 77 (1.5) |  | 14 (1.7) |
| Laborer |  | 253 (5.0) |  | 32 (3.9) |
| Business owner |  | 1400 (27.7) |  | 153 (18.5) |
| Private service |  | 199 (3.9) |  | 38 (4.6) |
| Other |  | 41 (0.8) |  | 9 (1.1) |
| *Household Characteristics* |  |  |  |  |
| Household Size | 5060 | 4.5 ± 1.9 | 736 | 4.45 ± 2.26 |
| Household head's occupation | 4952 |  |  |  |
| No occupation |  | 61 (1.2) | 723 | 9 (1.1) |
| Farmer/sharecropper |  | 700 (13.8) |  | 106 (12.8) |
| Laborer |  | 1642 (32.4) |  | 236 (28.5) |
| Business owner |  | 1670 (33.0) |  | 234 (28.3) |
| Private service |  | 756 (14.9) |  | 119 (14.4) |
| Other |  | 123 (2.4) |  | 19 (2.3) |
| Living standard index ^a^ | 5065 | -0.16  ± 0.92 | 738 | -0.15 ± 0.92 |
| Toilet | 5063 |  | 737 |  |
| None/field/bush |  | 212 (4.2) |  | 22 (2.7) |
| Open latrine |  | 1039 (20.4) |  | 162 (19.59) |
| Sealed or ventilated latrine |  | 3035 (59.9) |  | 436 (52.72) |
| Flush toilet |  | 777 (15.3) |  | 116 (14.03) |
| SSC: Secondary School Certificate  ^a^ Living standards index (LSI) was calculated using principal component analysis (PCA) from household assets and housing characteristics | | | | |
|  | | | | |

| **Supplemental Table 3**. The association between food environment indicators and food variety scores (using a 7 day recall period) among postpartum women who reported being able to buy small food purchases sometimes or always (n=3,108) | | | | | | |
| --- | --- | --- | --- | --- | --- | --- |
|  | **Model 1^a^** | | **Model 2^b^** | | **Model 3^c^** | |
|  | Estimate (95% CI) | p-value | Estimate (95% CI) | p-value | Estimate (95% CI) | p-value |
| Markets |  |  |  |  |  |  |
| Distance to nearest market (100m)^d^ | -0.04 (-0.07, -0.00) | 0.04 | -0.02 (-0.05, 0.01) | 0.26 | -0.02 (-0.05, 0.02) | 0.28 |
| Density of markets within 1600m^e^ |  |  |  |  |  |  |
| 0 to 2 markets | Ref |  | Ref |  | Ref |  |
| 3 to 4 markets | 0.37 (0.00, 0.74) | 0.05 | 0.43 (0.08, 0.77) | 0.01 | 0.46 (0.12, 0.81) | <0.01 |
| 5 to 6 markets | 0.63 (0.25, 1.01) | <0.01 | 0.56 (0.21, 0.91) | <0.01 | 0.61 (0.25, 0.97) | <0.001 |
| 7 ≥ markets | 0.78 (0.39, 1.17) | <0.001 | 0.71 (0.35, 1.07) | <0.001 | 0.81 (0.42, 1.21) | <0.001 |
| Grocery shops |  |  |  |  |  |  |
| Distance to nearest grocery shop (100m) | 0.09 (-0.01, 0.19) | 0.08 | 0.10 (0.01, 0.20) | 0.03 | 0.10 (0.01, 0.20) | 0.03 |
| Density of grocery shops within 400m |  |  |  |  |  |  |
| 0 to 1 grocery shops | Ref |  | Ref |  | Ref |  |
| 2 to 3 grocery shops | -0.64 (-1.05, -0.24) | <0.01 | -0.62 (-0.99, -0.25) | <0.01 | -0.62 (-1.00, -0.25) | <0.01 |
| 4 to 5 grocery shops | -0.21 (-0.61, 0.19) | 0.31 | -0.27 (-0.64, 0.10) | 0.16 | -0.27 (-0.64, 0.10) | 0.15 |
| 6 ≥ grocery shops | -0.43 (-0.81, -0.04) | 0.03 | -0.43 (-0.78, -0.07) | 0.02 | -0.45 (-0.80, -0.09) | 0.01 |
| Density of grocery shops within 1600m |  |  |  |  |  |  |
| 0 to 44 grocery shops | Ref |  | Ref |  | Ref |  |
| 45 to 54 grocery shops | -0.15 (-0.51, 0.21) | 0.41 | -0.11 (-0.44, 0.22) | 0.52 | -0.24 (-0.57, 0.10) | 0.17 |
| 55 to 67 grocery shops | 0.10 (-0.26, 0.46) | 0.59 | -0.02 (-0.35, 0.32) | 0.92 | -0.19 (-0.53, 0.16) | 0.29 |
| 68 ≥ grocery shops | -0.03 (-0.39, 0.33) | 0.87 | 0.01 (-0.32, 0.35) | 0.94 | -0.27 (-0.63, 0.10) | 0.15 |
| Food variety scores are defined as the average number of non-starchy staple foods items or groups consumed in the last week excluding sweet & salty snacks and sugary sweetened beverages. Scores range from 1-25. ^a^ Model 1 controls of maternal age ^b^ Model 2 controls for maternal age, living standards index (categorical), maternal education (categorical), number of people in household, and season of 3-month dietary recall (categorical) ^c^ Model 3 controls for model 2 covariates + distance/ density of grocery shops (market models) or markets (grocery shop models)  ^d^ Distance variables were scaled to 100m units. Negative numbers indicate households living further from food vendors have on average lower food variety scores.  ^e^ Density variables were categorized using quartiles based on distribution of data | | | | | | |

| **Supplemental Table 4.** The association between food environment indicators and dietary diversity scores among postpartum women (n=5,064). | | | | | | |
| --- | --- | --- | --- | --- | --- | --- |
|  | **Model 1^a^** | | **Model 2^b^** | | **Model 3^c^** | |
|  | IRR (95% CI) | p-value | IRR (95% CI) | p-value | IRR (95% CI) | p-value |
| Markets |  |  |  |  |  |  |
| Distance to nearest market (100m)^d^ | 1.00 (0.99, 1.00) | 0.15 | 1.00 (0.99, 1.00) | 0.38 | 1.00 (0.99, 1.00) | 0.39 |
| Density of markets within 1600m^e^ |  |  |  |  |  |  |
| 0 to 2 markets | Ref |  | Ref |  | Ref |  |
| 3 to 4 markets | 1.03 (0.99, 1.07) | 0.20 | 1.03 (0.99, 1.07) | 0.20 | 1.03 (0.99, 1.07) | 0.14 |
| 5 to 6 markets | 1.04 (0.99, 1.08) | 0.09 | 1.03 (0.99, 1.08) | 0.11 | 1.04 (1.00, 1.08) | 0.06 |
| 7 ≥ markets | 1.07 (1.02, 1.11) | <0.01 | 1.06 (1.02, 1.11) | <0.01 | 1.07 (1.03, 1.12) | <0.01 |
| Grocery shops |  |  |  |  |  |  |
| Distance to nearest grocery shop (100m) | 1.01 (0.99, 1.02) | 0.34 | 1.01 (0.99, 1.02) | 0.33 | 1.01 (0.99, 1.02) | 0.33 |
| Density of grocery shops within 400m |  |  |  |  |  |  |
| 0 to 1 grocery shops | Ref |  | Ref |  | Ref |  |
| 2 to 3 grocery shops | 0.98 (0.93, 1.02) | 0.27 | 0.98 (0.93, 1.02) | 0.28 | 0.98 (0.94, 1.02) | 0.28 |
| 4 to 5 grocery shops | 0.99 (0.95, 1.04) | 0.74 | 0.99 (0.95, 1.03) | 0.57 | 0.99 (0.95, 1.03) | 0.56 |
| 6 ≥ grocery shops | 0.97 (0.94, 1.02) | 0.22 | 0.97 (0.93, 1.01) | 0.17 | 0.97 (0.93, 1.01) | 0.15 |
| Density of grocery shops within 1600m |  |  |  |  |  |  |
| 0 to 44 grocery shops | Ref |  | Ref |  | Ref |  |
| 45 to 54 grocery shops | 1.00 (0.97, 1.04) | 0.84 | 1.00 (0.96, 1.04) | 0.93 | 0.99 (0.95, 1.03) | 0.69 |
| 55 to 67 grocery shops | 1.00 (0.96, 1.04) | 0.97 | 0.99 (0.95, 1.03) | 0.67 | 0.98 (0.94, 1.02) | 0.24 |
| 68 ≥ grocery shops | 1.00 (0.96, 1.04) | 0.97 | 1.00 (0.96, 1.04) | 0.97 | 0.97 (0.93, 1.02) | 0.21 |
| Dietary diversity scores were defined as number of food groups consumed at least 3 times on average in the 7-day recall period. Scores range from 1-10. Food groups defined using MDDW guidelines: cereals, flesh foods, pulses & beans, dark leafy green vegetables, other vitamin A-rich vegetables, dairy, eggs, other vegetables, other fruits, and nuts & seeds.  ^a^ Model 1 controls of maternal age ^b^ Model 2 controls for maternal age, living standards index (categorical), maternal education (categorical), number of people in household, and season of 3-month dietary recall (categorical) ^c^ Model 3 controls for model 2 covariates + distance/ density of grocery shops (market models) or markets (grocery shop models)  ^d^ Distance variables were scaled to 100m units. Negative numbers indicate households living further from food vendors have on average lower food variety scores.  ^e^ Density variables were categorized using quartiles based on distribution of data | | | | | | |

| **Supplemental Table 5.** The association between food environment indicators and dietary diversity scores among postpartum women who reported being able to buy small food purchases sometimes or always (n=3,108) | | | | | | |
| --- | --- | --- | --- | --- | --- | --- |
|  | **Model 1^a^** | | **Model 2^b^** | | **Model 3^c^** | |
|  | IRR (95% CI) | p-value | IRR (95% CI) | p-value | IRR (95% CI) | p-value |
| Markets |  |  |  |  |  |  |
| Distance to nearest market (100m)^d^ | 1.00 (0.99, 1.00) | 0.16 | 1.00 (0.99, 1.00) | 0.40 | 1.00 (0.99, 1.00) | 0.42 |
| Density of markets within 1600m^e^ |  |  |  |  |  |  |
| 0 to 2 markets | Ref |  | Ref |  | Ref |  |
| 3 to 4 markets | 1.03 (0.98, 1.08) | 0.32 | 1.03 (0.98, 1.08) | 0.23 | 1.04 (0.99, 1.09) | 0.17 |
| 5 to 6 markets | 1.05 (0.99, 1.10) | 0.09 | 1.04 (0.99, 1.09) | 0.14 | 1.05 (0.99, 1.10) | 0.09 |
| 7 ≥ markets | 1.07 (1.02, 1.13) | 0.01 | 1.06 (1.01, 1.12) | 0.02 | 1.07 (1.02, 1.14) | 0.01 |
| Grocery shops |  |  |  |  |  |  |
| Distance to nearest grocery shop (100m) | 1.01 (0.99, 1.02) | 0.28 | 1.01 (1.00, 1.02) | 0.21 | 1.01 (0.99, 1.02) | 0.22 |
| Density of grocery shops within 400m |  |  |  |  |  |  |
| 0 to 1 grocery shops | Ref |  | Ref |  | Ref |  |
| 2 to 3 grocery shops | 0.96 (0.91, 1.01) | 0.14 | 0.96 (0.91, 1.02) | 0.17 | 0.96 (0.91, 1.02) | 0.16 |
| 4 to 5 grocery shops | 0.97 (0.92, 1.03) | 0.32 | 0.97 (0.92, 1.02) | 0.26 | 0.97 (0.92, 1.02) | 0.25 |
| 6 ≥ grocery shops | 0.96 (0.91, 1.01) | 0.12 | 0.96 (0.91, 1.01) | 0.12 | 0.96 (0.91, 1.01) | 0.10 |
| Density of grocery shops within 1600m |  |  |  |  |  |  |
| 0 to 44 grocery shops | Ref |  | Ref |  | Ref |  |
| 45 to 54 grocery shops | 0.99 (0.94, 1.04) | 0.68 | 0.99 (0.95, 1.04) | 0.74 | 0.98 (0.93, 1.03) | 0.45 |
| 55 to 67 grocery shops | 0.99 (0.94, 1.04) | 0.66 | 0.98 (0.93, 1.03) | 0.40 | 0.97 (0.92, 1.01) | 0.17 |
| 68 ≥ grocery shops | 1.00 (0.95, 1.05) | 0.96 | 1.00 (0.96, 1.05) | 0.91 | 0.98 (0.93, 1.03) | 0.39 |
| IRR: Incidence Rate Ratios, CI: Confidence Intervals Dietary diversity scores were defined as number of food groups consumed at least 3 times on average in the 7-day recall period. Scores range from 1-10. Food groups defined using MDDW guidelines: cereals, flesh foods, pulses & beans, dark leafy green vegetables, other vitamin A-rich vegetables, dairy, eggs, other vegetables, other fruits, and nuts & seeds.  ^a^ Model 1 controls of maternal age ^b^ Model 2 controls for maternal age, living standards index (categorical), maternal education (categorical), number of people in household, and season of 3-month dietary recall (categorical) ^c^ Model 3 controls for model 2 covariates + distance/ density of grocery shops (market models) or markets (grocery shop models)  ^d^ Distance variables were scaled to 100m units. Negative numbers indicate households living further from food vendors have on average lower food variety scores.  ^e^ Density variables were categorized using quartiles based on distribution of data | | | | | | |

| **Supplemental Table 6.** The association between food environment indicators and consumption of less healthy food options (≥ 3 times over 7 days) among postpartum women who reported being able to buy small food purchases sometimes or always (n=3,108) | | | | | | |
| --- | --- | --- | --- | --- | --- | --- |
|  | **Model 1^a^** | | **Model 2^b^** | | **Model 3^c^** | |
|  | Odds Ratio | p-value | Odds Ratio | p-value | Odds Ratio | p-value |
|  | (95% CI) |  | (95% CI) |  | (95% CI) |  |
| Markets |  |  |  |  |  |  |
| Distance to nearest market (100m)^d^ | 0.98 (0.96, 1.00) | 0.03 | 0.98 (0.96, 1.00) | 0.08 | 0.98 (0.96, 1.00) | 0.08 |
| Density of markets within 1600m^e^ |  |  |  |  |  |  |
| 0 to 2 markets | Ref |  | Ref |  | Ref |  |
| 3 to 4 markets | 1.01 (0.83, 1.25) | 0.89 | 1.06 (0.85, 1.31) | 0.61 | 1.08 (0.87, 1.34) | 0.49 |
| 5 to 6 markets | 1.14 (0.92, 1.40) | 0.23 | 1.12 (0.90, 1.39) | 0.33 | 1.15 (0.92, 1.43) | 0.23 |
| 7 ≥ markets | 1.01 (0.81, 1.25) | 0.95 | 0.98 (0.78, 1.23) | 0.84 | 1.02 (0.80, 1.31) | 0.88 |
| Grocery shops |  |  |  |  |  |  |
| Distance to nearest grocery shop (100m) | 1.05 (1.00, 1.11) | 0.07 | 1.06 (1.00, 1.13) | 0.04 | 1.06 (1.00, 1.13) | 0.04 |
| Density of grocery shops within 400m |  |  |  |  |  |  |
| 0 to 1 grocery shops | Ref |  | Ref |  | Ref |  |
| 2 to 3 grocery shops | 0.96 (0.77, 1.20) | 0.71 | 0.98 (0.77, 1.23) | 0.83 | 0.97 (0.77, 1.23) | 0.83 |
| 4 to 5 grocery shops | 0.90 (0.72, 1.12) | 0.35 | 0.88 (0.70, 1.12) | 0.30 | 0.88 (0.70, 1.12) | 0.30 |
| 6 ≥ grocery shops | 0.93 (0.75, 1.15) | 0.50 | 0.93 (0.75, 1.16) | 0.53 | 0.92 (0.74, 1.15) | 0.48 |
| Density of grocery shops within 1600m |  |  |  |  |  |  |
| 0 to 44 grocery shops | Ref |  | Ref |  | Ref |  |
| 45 to 54 grocery shops | 1.00 (0.82, 1.21) | 0.97 | 1.01 (0.82, 1.24) | 0.92 | 1.00 (0.81, 1.24) | 0.99 |
| 55 to 67 grocery shops | 0.91 (0.74, 1.11) | 0.34 | 0.85 (0.69, 1.05) | 0.14 | 0.84 (0.68, 1.05) | 0.12 |
| 68 ≥ grocery shops | 0.92 (0.75, 1.12) | 0.41 | 0.91 (0.74, 1.13) | 0.40 | 0.92 (0.73, 1.16) | 0.49 |
| Less healthy food consumption is defined as consumption of less healthy food options at least 3 times on average in the 7-day recall period. Less healthy foods options were defined as soda, sweet yogurt, sugar cane, cake/biscuits, mishti, chocolate/candy, ice cream, salty snacks,and food fried in oil ^a^ Model 1 controls of maternal age ^b^ Model 2 controls for maternal age, living standards index (categorical), maternal education (categorical), number of people in household, and season of 3-month dietary recall (categorical)  ^c^ Model 3 controls for model 2 covariates + distance/ density of grocery shops (market models) or markets (grocery shop models)  ^d^ Distance variables were scaled to 100m units. Negative numbers indicate households living further from food vendors have on average lower food variety scores.  ^e^ Density variables were categorized using quartiles based on distribution of data | | | | | | |
